# Dynamic Adsorption/Desorption of NO_x_ on MFI Zeolites: Effects of Relative Humidity and Si/Al Ratio

**DOI:** 10.3390/nano13010156

**Published:** 2022-12-29

**Authors:** Haiyang Tao, Yingshu Liu

**Affiliations:** 1 School of Energy and Environmental Engineering, University of Science and Technology Beijing, Beijing 100083, China; 2 Beijing Higher Institution Engineering Research Center of Energy Conservation and Environmental Protection, Beijing 100083, China

**Keywords:** flue gas, MFI zeolites, NO_x_ adsorption, Si/Al, water vapor

## Abstract

Adsorption is a potential technology that is expected to meet NO_x_ ultra-low emission standards and achieve the recovery of NO_2_. In this study, the adsorption/desorption behavior of NO_x_ with competitive gases (e.g., H_2_O(g) and CO_2_) was studied on MFI zeolites with different Si/Al ratios and under different relative humidity (0~90% RH). Sample characterization of self-synthesizing zeolites was conducted by means of X-ray diffraction, Ar adsorption-desorption, and field emission scanning electron microscopy. The results showed that low-silica HZSM-5(35) showed the highest NO_x_ adsorption capacity of 297.8 μmol/g (RH = 0) and 35.4 μmol/g (RH = 90%) compared to that of other adsorbents, and the efficiency loss factor of NO_x_ adsorption capacity at 90%RH ranged from 85.3% to 88.1%. A water-resistance strategy was proposed for NO_x_ multicomponent competitive adsorption combined with dynamic breakthrough tests and static water vapor adsorption. The presence of 14% O_2_ and lower adsorption temperature (25 °C) favored NO_x_ adsorption, while higher CO_2_ concentrations (~10.5%) had less effect. The roll-up factor (*η*) was positively correlated with lower Si/Al ratios and higher H_2_O(g) concentrations. Unlike Silicalite-1, HZSM-5(35) exhibited an acceptable industrial desorption temperature window of NO_2_ (255~265 °C). This paper aims to provide a theoretical guideline for the rational selection of NO_x_ adsorbents for practical applications.

## 1. Introduction 

The vast emissions of nitrogen oxides (NO_x_, x = 1, 2) have caused deleterious effects on human health and the ecological environment, including acid rain, photochemical smog, and ozone layer depletion, etc. [1]. The strictest ultra-low emission standards (e.g., NO_x_ ≤ 50 mg/m^3^) have been promulgated since 2019 [2]. Many attempts have been made for the efficient elimination of NO_x_ including reduction [3], oxidation [4,5,6], decomposition [7], and adsorption technologies [8]. Selective catalytic reduction (SCR), as the most well-established deNO_x_ technology, can convert toxic NO_x_ into harmless N_2_. In fact, NO_x_ is not worthless. NO, as a therapeutic agent, can prevent thrombosis [9]. High-purity NO_2_, as the main source of bulk chemicals (e.g., nitric acid and nitrogen fertilizer), sells for 6000 USD/ton in the Chinese market [10,11]. Fortunately, the adsorption technology can fulfill the requirements of NO_x_ deep purification (<1 ppm) and non-destructive NO_2_ recovery.

Adsorbents are the key to adsorption technology. Various NO_x_ adsorbents have been screened such as activated carbons (ACs), metal-organic frameworks (MOFs), polyoxometalates (POMs), and zeolites. However, two thorny issues need to be addressed. On one hand, adsorbents can either strongly adsorb NO (~95% of NO_x_) or NO_2_ from the efficient oxidation of NO. There is no doubt that NO adsorption, as a supercritical gas, is more challenging than NO_2_ due to the low boiling point of NO (−152 °C) [12]. On the other hand, the concentrations of H_2_O(g) in real flue gas are several orders of magnitude higher than NO_x_ resulting in preferential adsorption of strong polarity H_2_O(g) and thereby severely influencing the effectiveness of the adsorption process even the structural collapse of the adsorbents [13]. Recently, Guo et al. [14] found that NO cannot be oxidized to NO_2_ for pitch-ACF at 20% RH. Similarly, the follow-up report reconfirmed that the conversion of NO sharply drops to 0% at 50% RH [15]. DeCoste et al. [16] found that Mg-MOF-74 was completely collapsed upon exposure to humid conditions, which is responsible for the hydroxyl group in water vapor attacking relatively weak metal-oxygen coordination. Wang et al. [17] reported that the NO_x_ adsorption capacity decreased for HGeW polyoxometalate after three regeneration cycles. Contrastingly, zeolites, as typical crystalline aluminosilicates with well-defined microporosities, have promising applications for the treatment of flue gas denitration benefiting from appealing features such as highly ordered aperture size, tunable hydrophilic-hydrophobic, and excellent thermal stability [18]. Recently, studies on NO_x_ adsorption over zeolites have been reported under dry conditions. The order of NO adsorption capacity modified by acid treatment and ion-exchanged MOR zeolites is Ni-MOR > Cu-MOR > Mn-MOR > Na-MOR [19]. Ca-beta zeolite exhibits a multifold increase in NO_x_ adsorption capacity from 0.1 to 221 μmol/g, and O_2_ plays a dominant role in the conversion of NO and physisorption of NO_2_ [20]. Hu et al. [21] found that efficient conversion of NO on MFI zeolites follows H^+^-ZSM-5(44%) > NH_4_^+^-ZSM-5(39%) > Na^+^-ZSM-5(36%). Liu et al. [22] reported a novel cyclic adsorption process and obtained concentrated NO_2_ by cryogenic condensation using MFI zeolite, exhibiting its reactive and oxidative nature.

Adjusting the Si/Al ratios of zeolites is an efficient hydrophobic strategy. The high silicon content in the framework of zeolites imparts strong hydrophobicity, causing a decrease in the affinity of H_2_O(g). Yin et al. [23] reported that high-temperature hydrothermal dealuminated NaY zeolite showed superior hydrophobic performance with ~95% H_2_O(g) being blocked at 50% RH compared with the pristine zeolite. Adsorptive removal of dichloromethane was performed on MFI zeolites with different Si/Al ratios, wherein ZSM-5(200) with the highest Si/Al ratio exhibited a robust adsorption performance and hydrophobicity [24]. However, few studies have focused on the NO_x_ adsorption/desorption behavior of zeolites. 

In this work, a detailed compilation of competitive adsorption behavior containing NO_x_-H_2_O(g)-CO_2_ multicomponent gases was studied on MFI zeolites with different Si/Al ratios under different RH. Further, multiple influential factors, including O_2_ concentrations, CO_2_ concentrations, and adsorption temperatures, were systematically investigated by the dynamic breakthrough tests to fully get valuable insights into NO_x_ adsorption under 90%RH. Meanwhile, the temperature-programmed desorption (TPD) of NO_x_ and regeneration tests were studied. 

## 2. Materials and Methods

### 2.1. Synthesis of Materials 

In each synthesis of MFI zeolites with different Si/Al ratios, a certain amount of sodium aluminate (NaAlO_2_) and sodium hydroxide (NaOH) was dissolved in deionized water until it became clear under stirring at 500 rpm for 0.5 h. After that, tetraethyl orthosilicate (TEOS) and tetrapropylammonium hydroxide (TPAOH) were added dropwise into the resulting mixture and stirred at 500 rpm for 1 h with the final molar ratio of 5.9Na_2_O:100SiO_2_:xAl_2_O_3_:1256H_2_O:40.4TPAOH (x = 2.85, 0.91, 0.28, 0). Finally, the obtained gel was transferred into an autoclave and placed in an oven at crystallization temperature (170 °C) for 72 h. After centrifugation and repeated rinsing with deionized water, samples were dried overnight at 110 °C and calcined at 550 °C for 5 h. Afterward, the as-made slurry Na^+^-type samples were dispersed with a 1 M ammonium chloride (NH_4_Cl) aqueous solution at 80 °C for 12 h under stirring and by reflux. The ion exchange process was repeated at least three times to guarantee the full ammoniacal zeolite. NH_4_^+^-type samples were separated by filtration again, dried at 110 °C overnight, and calcined in a muffle furnace at 550 °C for 5 h. The as-synthesized samples with different Si/Al ratios were hereinafter referred as HZSM-5(*X*) (*X* = 35, 110, and 360), and Silicalite-1 (pure silica MFI zeolite), respectively.

### 2.2. Characterization

Powder X-ray diffraction patterns (PXRD) were characterized using a Bruker D8 Advance (Germany) equipped with Cu-Kα radiation (λ = 0.154 nm) operated at 40 kV and 40 mA. XRD patterns were taken over the range from 5° to 60° at a scanning speed of 5°/min. Ar physisorption isotherms were measured at −186 °C (Quantachrome, Boynton Beach, FL, USA), where the pore size distribution was calculated according to the nonlocal density functional theory (NLDFT) model, and the total pore volume was calculated at P/P_0_ = 0.99. The specific surface areas were calculated based on the Brunauer–Emmett–Teller (BET) method from the adsorption branches of the isotherms with the P/P_0_ range from 0.05 to 0.25. The microporous surface areas and external surface areas were calculated using the t-plot method. The mesoporous surface areas were calculated by the Barrett–Joyner–Halenda (BJH) method from the desorption branches of the isotherms. All samples were activated at 300 °C for 12 h. The information on surface morphological characteristics was observed via high-resolution field emission scanning electron microscopy (Hitachi SU8020 UHR, Tokyo, Japan). All samples were sputtered with platinum before imaging. The static water vapor adsorption isotherms were measured at 25 °C using a 3H-2000PW gravimetry vapor adsorption analyzer instrument (BeiShiDe, Beijing, China). Prior to adsorption, all samples were degassed under vacuum at 300 °C for 12 h to remove impurities. 

### 2.3. Dynamic Adsorption–Desorption Tests

**Figure 1:**
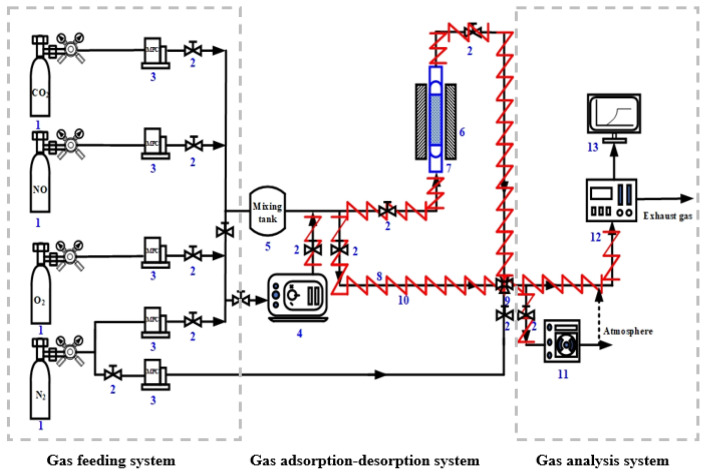
Schematic diagram of NO_x_ adsorption-desorption experimental setup, with the components: 1—gas cylinder, 2—ball valve, 3—mass flow controller, 4—steam generator, 5—mixing tank, 6—furnace, 7—adsorption column, 8—bypass line, 9—four-way valve, 10—electric heating belts (red), 11—hygrometer, 12—flue gas analyzer, and 13—data collection device.

The NO_x_ adsorption-desorption tests of all samples were performed by a self-made dynamic breakthrough experimental setup containing three parts (i.e., a gas feeding system, a gas adsorption-desorption system, and a gas analysis system), as shown in Figure 1. The feed gas contained 200 ppm NO_x_, 14% O_2_, 4.5% CO_2_, and the carrier gas N_2_. N_2_ was divided into two branches: one was to ensure the normal operation of the flue gas analyzer in the bypass line (flow rate of 750 mL/min), and the other was to adjust the H_2_O(g) concentrations required in the steam generator (flow rate of 250 mL/min) to eventually merge into the adsorption column with upstream gases mixed simultaneously. Temperature-controlled electric heating belts marked in red were used to prevent H_2_O(g) condensation. Then, ~2.25 g of pelletized samples (40~60 meshes) were loaded into a vertical quartz adsorption column (internal diameter of 6 mm and column length of 20 cm) capped with quartz wool on both sides. Before the adsorption tests, samples were in situ activated under the flow of N_2_ (50 mL/min) from 25 °C to 550 °C (10 °C/min) and held 550 °C for 1 h. The gas analysis system included a flue gas analyzer (MRU, Vario Plus, Obereisesheim, Germany) and a hygrometer (Rotronic, HC2A-S, Bassersdorf, Switzerland) that can record outlet concentrations of H_2_O(g), NO, NO_2_, and NO_x_. 

The adsorption capacity of NO_x_ was calculated by the following formula [21].

(1) $$\;q_{e}=\frac{{[C}_{in}\times t-\int_{0}^{t}C_{out}dt]\times F}{{m\times V}_{m}}$$

where *q_e_* is the adsorption capacity for NO_x_, in mmol/g; *F* is the flue gas flow rate, in mL/min; *m* is the weight of adsorbents, in g; *C_in_* and *C_out_* represent the inlet and outlet concentrations of NO_x_, respectively, in ppm; *V_m_* is the molar volume of the gas, 24.5 L/mol (25 °C, 1 atm); The breakthrough and saturation adsorption capacity of NO_x_ is determined when *C_out_/C_in_* reaches 0.05 and 0.95, respectively.

Temperature-programmed desorption (TPD) tests were conducted once the sample was completely saturated. Prior to each desorption test, a ~20 min stabilization period by purging N_2_ (1 L/min) in the bypass line was conducted to remove the interference from H_2_O(g) and other NO_x_ products until the detector returned to its initial state. The desorption temperature procedure increased from 25 to 550 °C with a heating rate of 10 °C/min and held at 550 °C for 1 h. Meanwhile, six consecutive regeneration tests were conducted at 90% RH.

## 3. Results and Discussion

### 3.1. Physical Characteristics and Surface Morphology

**Figure 2:**
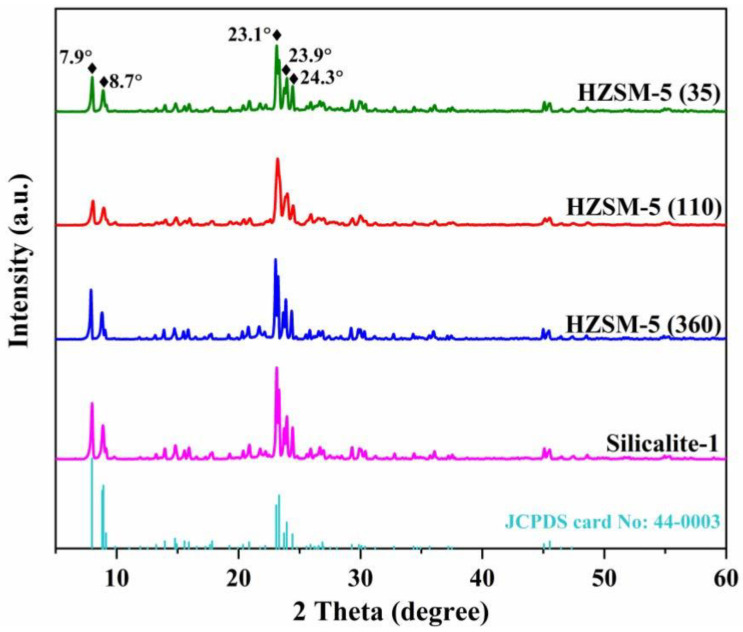
XRD patterns of all samples.

**Figure 3:**
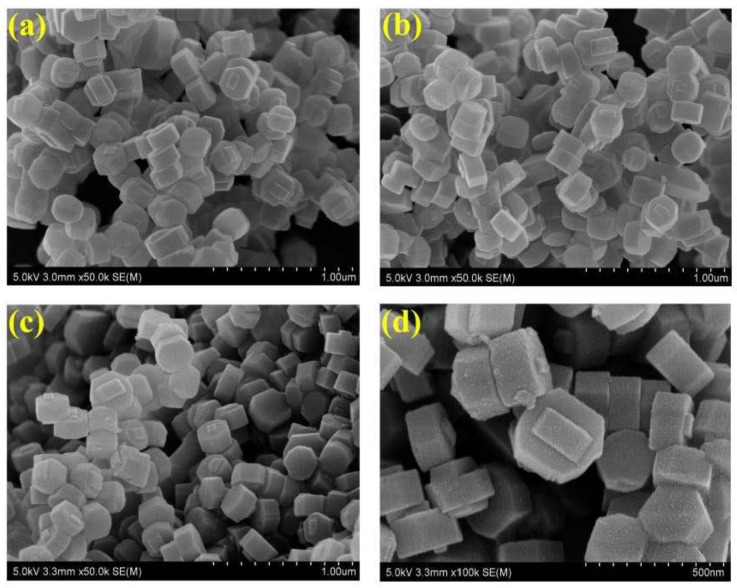
SEM images of all samples (**a**) HZSM-5(35), (**b**) HZSM-5(110), (**c**) HZSM-5(360), and (**d**) Silicalite-1.

Figure 2 shows the XRD patterns of the as-synthesized samples with the major diffraction peaks at 2θ of 7.9°, 8.7°, 23.1°, 23.9°, and 24.3°, implying the successful synthesis of the MFI framework topologies (JCPDS card No. 44-0003) [25]. The diffraction peaks of all MFI samples indicate that adjusting the Si/Al ratios has no effect on the zeolite framework. The information on morphological features and crystal sizes is displayed by SEM images. As can be seen in Figure 3, all the crystal particles tend to be loosely stacked, but local agglomeration occurs due to high surface Gibbs energy [26]. Each crystal particle presents regular hexagonal with uniform average crystal sizes approximately in the range of 200~300 nm along the c-axis direction in each sample. It can be seen from Figure 3a–d that the surfaces of the crystals gradually become coarse, which is responsible for the rapid nucleation and surface etching under high alkaline conditions [27]. The evolution of the convex-concave surfaces of all samples becomes more evident, resulting in the formation of multiscale surface roughness and enhancing the hydrophobicity [28].

**Figure 4:**
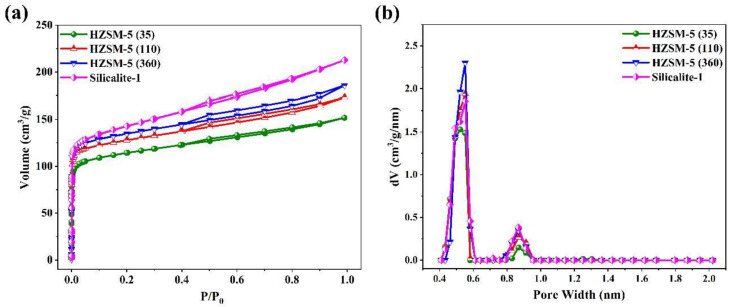
(**a**) Ar adsorption-desorption isotherms of all samples. (**b**) Pore size distribution of all samples.

Ar adsorption-desorption isotherms and pore size distributions of all MFI samples are shown in Figure 4, and the corresponding textural parameters including special surface areas, pore volumes, and average pore sizes are listed in Table 1. As shown in Figure 4a, the steep Ar uptake at very low pressure (P/P_0_ < 0.1) is observed in type I isotherms featuring typical microporous structures, which is ascribed to the strong interaction between adsorbent and adsorbate, i.e., the microporosity is filled [29]. A significant adsorbed amount increases due to the intercrystalline voids and high surface roughness at the intermediate pressure of P/P_0_ = 0.4~0.9 [30]. Moreover, no obvious hysteresis loop at P/P_0_ > 0.9 is observed, suggesting that no aggregated mesopores are formed [31]. The BET special surface areas and total pore volumes gradually increase with increasing Si/Al ratios. The main reason is that there is no non-framework aluminum formed. According to Figure 4b, the average pore sizes of the samples are mostly distributed in the range from 0.52 to 0.55 nm. Almost no aluminum atoms are arranged in the zeolite framework for Silicalite-1, which leads to the unit cell dimensions shrinking and a decrease in average pore size due to the difference in bond lengths (i.e., the Si-O and Al-O bond lengths of 1.64 Å and 1.75 Å, respectively) [32,33].

### 3.2. Static Adsorption of Water Vapor 

**Figure 5:**
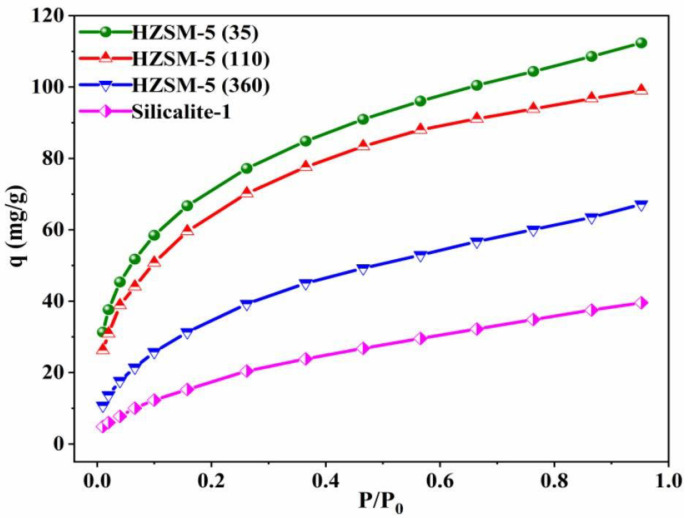
Water vapor adsorption isotherms on all samples at 25 °C.

The water vapor isotherms are displayed in Figure 5 and the resistance to water vapor is reflected in the weakened interaction between MFI zeolites and water vapor with increasing Si/Al ratios. Among them, Silicalite-1 has the strongest hydrophobicity with 37.4 mg/g water vapor uptake adsorbed at P/P_0_ = 0.9, which is consistent with the above discussion, i.e., increasing the surface roughness of zeolites enhances hydrophobicity. It also emphasizes that the amount of compensating cations in the zeolite framework greatly determines the water vapor uptake, and the reduction of the cations leads to a decrease in the strength of the electrostatic force, which in turn increases the van der Waals force [34].

### 3.3. Dynamic Adsorption of NO_x_

#### 3.3.1. Effect of Relative Humidity on NO_x_ Adsorption by MFI Zeolites with Different Si/Al Ratios

**Figure 6:**
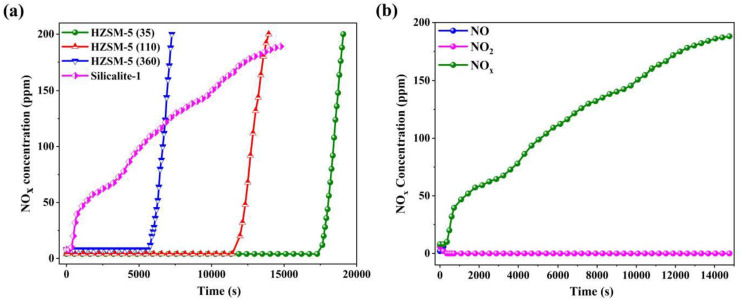
(**a**) Breakthrough curves of NO_x_ on all samples under dry conditions. (**b**) Breakthrough curves of NO, NO_2_, and NO_x_ on Silicalite-1.

Figure 6a shows the NO_x_ breakthrough curves of all MFI zeolites under dry conditions, and a significant downward trend in the NO_x_ saturation adsorption capacity is presented with increasing Si/Al ratios (e.g., HZSM-5(35) (297.8 μmol/g), HZSM-5(110) (206.8 μmol/g), HZSM-5(360) (96.5 μmol/g), and Silicalite-1 (59.2 μmol/g), respectively). HZSM-5(35) has a deep adsorption purification with the breakthrough and saturation time of 17,775 s and 19,040 s (i.e., accounting for ~94.3%), respectively, which shows the synergistic effect of NO oxidation and NO_2_ physisorption enhanced by more catalytic and adsorption sites. Silicalite-1 shows poor deep purification with the shortest breakthrough time of 504 s, yet exhibits a slower upward trend of NO_x_ with the saturation time of 14,040 s (i.e., accounting for ~3.6%). Figure 6b shows that NO is preferentially saturated, resulting in NO_2_ not being readily adsorbed and corroborating the strong dependence of NO_2_ on low-silica zeolite. 

**Figure 7:**
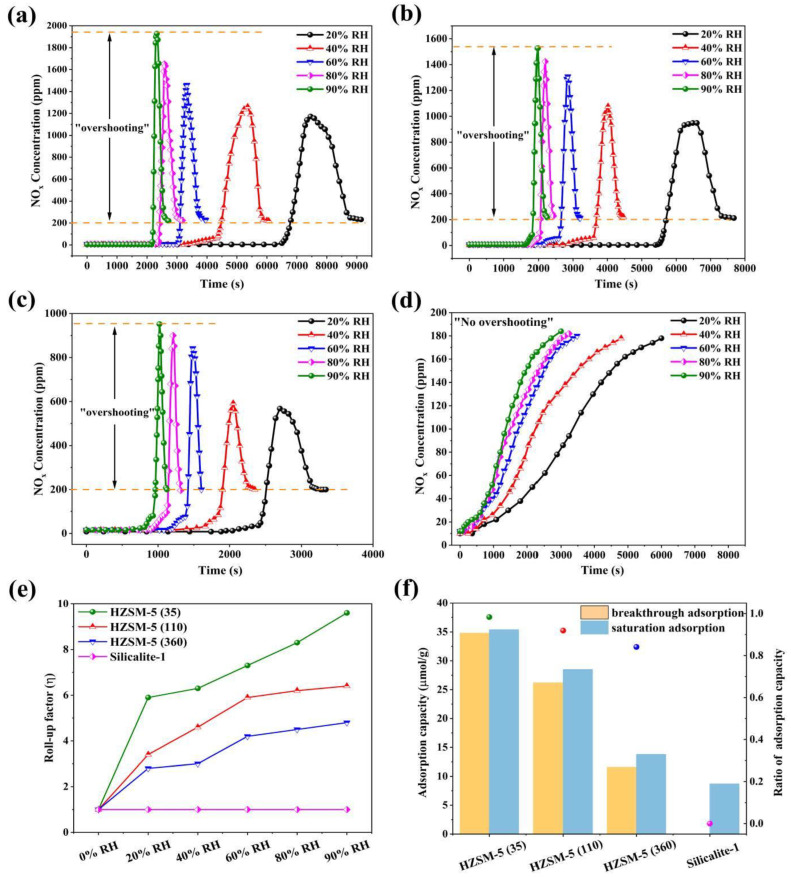
Breakthrough curves of NO_x_ under different RH conditions (**a**) HZSM-5(35), (**b**) HZSM-5(110), (**c**) HZSM-5(360), (**d**) Silicalite-1, (**e**) Roll-up factor and (**f**) Ratio of adsorption capacity.

To further demonstrate the competitive adsorption behavior of multicomponent gases (NO_x_-H_2_O(g)-CO_2_) on MFI zeolites, the dynamic adsorption tests were conducted using breakthrough tests under different RH conditions (20%, 40%, 60%, 80%, and 90%). Visibly, it can be seen from Figure 7a–d that HZSM-5(35) has the best NO_x_ adsorption performance, and the sequence of the NO_x_ adsorption capacities follows HZSM-5(35) > HZSM-5(110) > HZSM-5(360) > Silicalite-1, wherein the saturation adsorption capacity of NO_x_ on HZSM-5(35) dramatically decreases from 297.8 μmol/g (RH = 0) to 35.4 μmol/g (RH = 90%). For ease of comparison, the efficiency loss factor (*γ*) can be defined by the 1-(Q_humid_/Q_dry_), as summarized in Table 2. All adsorbents cumulatively drop by 88.1%, 86.2%, 85.7%, and 85.3%, respectively, which indicates that more NO_x_ adsorption sites are significantly dominated by the strong polarity of H_2_O(g). Silicalite-1 exhibits the strongest hydrophobicity by the static water vapor adsorption, yet exhibits the weakest NO_x_ competitive adsorption performance, which breaks the conventional thinking that increasing the Si/Al ratios enhance the hydrophobicity (i.e., VOCs, CO_2_, and N_2_/O_2_). In addition, it can also be directly proved the specificity of water-resistance rather than hydrophobicity for the NO_x_ competitive adsorption of multi-component gases.

Multiple overshooting peaks are presented in Figure 7a–d. The outlet NO_x_ concentrations show a multifold increase in comparison with the inlet concentrations. This peculiar phenomenon is called the roll-up effect and is characterized by the overshooting peak due to the competition between weakly adsorbed NO_x_ and strongly adsorbed H_2_O(g), thus yielding NO_2_-enriched gas. The roll-up factor (*η*) formula is as follows [35]:

(2) $$\eta =\frac{C_{out}}{C_{in}}$$

where *C_out_* is the maximum NO_x_ outlet concentration, and *C_in_* is the NO_x_ inlet concentration.

The *η* for HZSM-5(35) is 5.9, 6.3, 7.3, 8.3, and 9.6 as RH rises, and similar results are also presented for HZSM-5(110) and HZSM-5(360), as shown in Figure 7e. The maximum value of NO_x_ overshooting peak corresponds to the highest yield of NO_2_, which shows great potential for the highly concentrated NO_2_ from flue gases using the maximum roll-up factor. Additionally, the retention time of roll-up is gradually shortened, which is also responsible for the strong displacement interaction of H_2_O(g), which accelerates the adsorption competitive behavior. However, Silicalite-1 exhibits a lower NO_x_ adsorption capacity of barely 8.7 μmol/g at 90% RH. A conspicuous difference is observed that NO is preferentially saturated regardless of dry and humid conditions. Interestingly, the roll-up effect unexpectedly disappears for Silicalite-1. The reason could be ascribed to: (i) the poor NO conversion forming a trade-off between NO and NO_2_, (ii) the breakthrough time of H_2_O(g) is close to or essentially identical to that of NO_x_ [35], and (iii) the van der Waals forces play a dominant role [36]. It can be concluded that the positive correlations of *η* depend on the lower Si/Al ratios and higher H_2_O(g) concentrations. Figure 7f shows the ratio of breakthrough and saturation adsorption capacity of NO_x_ on MFI zeolites. The breakthrough adsorption capacity represents compliance with NO conversion efficiency, and low-silica zeolite HZSM-5(35) exhibits the highest ratio value (i.e., 0.98, 0.92, 0.84, and 0) compared to others with higher Si/Al ratio.

#### 3.3.2. Effect of O_2_ on NO_x_ Adsorption

**Figure 8:**
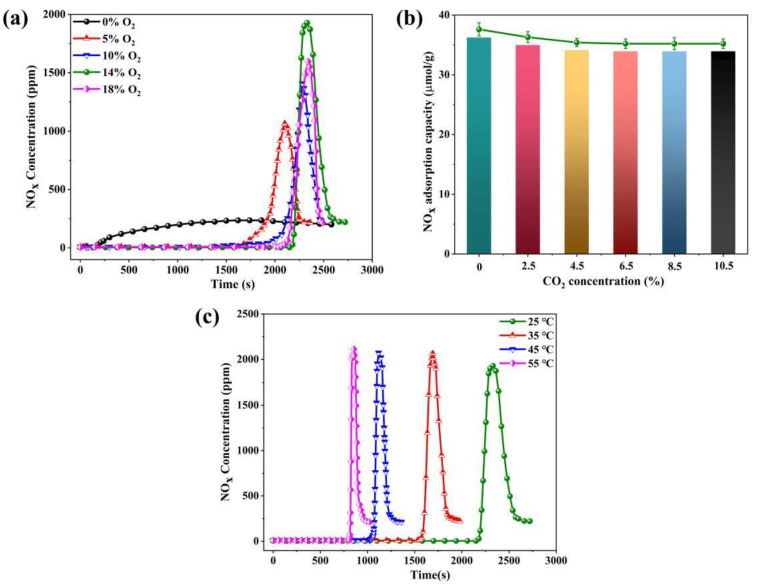
(**a**) Effect of O_2_ concentrations, (**b**) effect of CO_2_ concentrations, and (**c**) effect of different adsorption temperatures on NO_x_ adsorption of HZSM-5(35) at 90% RH.

To explore whether O_2_ concentrations affect NO_x_ adsorption at 90% RH, Figure 8a shows that NO is adsorbed and rapidly saturated in the absence of O_2_, with 6.8 μmol/g adsorption capacity. The adsorption capacity of NO_x_ gradually increases with the O_2_ concentrations from 5% to 18%. Further, an optimal concentration of 14%O_2_ is screened with the NO_x_ adsorption capacity of 35.4 μmol/g, which is conducive to shifting the equilibrium to the positive reaction direction. It indicates a dynamic adsorption equilibrium process of NO_x_ is established. In addition, the presence of O_2_ strengthens the conversion of NO and simultaneously accelerates the physisorption of NO_2_, which is an essential preceding step of NO_x_ adsorption. The increase in NO_x_ adsorption capacity is mainly due to the contribution of NO_2_, which reconfirms that NO_2_ is more easily adsorbed on HZSM-5(35) compared with NO. NO alone cannot exist a roll-up phenomenon, which is responsible for the low solubility of NO in H_2_O(g) based on Henry’s law [37]. The maximum *η* is 9.6 in the presence of 14% O_2_ and excessive O_2_ concentration (18%) inhibits NO_x_ adsorption and *η* decreases to 8.2.

#### 3.3.3. Effect of CO_2_ on NO_x_ Adsorption 

The flue gas contains large amounts of CO_2_, of which the concentration of CO_2_ is several orders of magnitude higher than the NO_x_ concentrations. The relationship of NO_x_ adsorption with various concentrations of CO_2_ on HZSM-5(35) at 90% RH (Figure 8b). The NO_x_ adsorption capacity is 37.2 μmol/g without CO_2_ while the NO_x_ adsorption capacity slightly decreases by 5.2% until the CO_2_ concentration reaches 6.5%. Furthermore, the NO_x_ adsorption capacity remains essentially unaffected even at higher CO_2_ concentrations, suggesting that the adsorption sites can be occupied by strong polarity H_2_O(g) and NO_x_. Overall, it is considered that CO_2_ has a negligible effect on NO_x_ adsorption. 

#### 3.3.4. Effect of Temperature on NO_x_ Adsorption

To further explore the effect of temperatures on NO_x_ adsorption, Figure 8c shows the NO_x_ breakthrough curves on HZSM-5(35) at 90% RH. The NO_x_ adsorption capacity is 35.4 μmol/g (25 °C), 25.1 μmol/g (35 °C), 16.5 μmol/g (45 °C), and 12.6 μmol/g (55 °C) at 90% RH, which decreases by 29.1%, 53.4%, and 64.4%. On one hand, adsorption is an exothermic reaction due to the negative activation energy [38,39]. On the other hand, lower NO conversion cannot be susceptible to the formation of NO_2_ at higher temperatures [40,41]. Figure 8c shows the *η* increases from 9.6 to 10.6 as the temperatures increase. Moreover, the retention time of the roll-up phenomenon corresponding to the adsorption temperature is 520 s, 380 s, 290 s, and 240 s, which decreases by 27.1%, 44.7%, and 60.3%, respectively. It elucidates that faster adsorption kinetics are dominated by the higher H_2_O(g) concentrations and lower adsorption temperatures.

### 3.4. Temperature-Programmed Desorption of NO_x_

**Figure 9:**
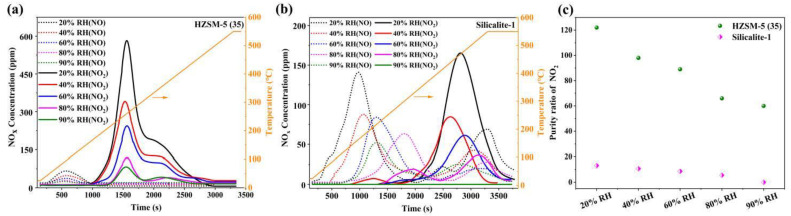
TPD curves of NO_x_ under different RH conditions: (**a**) HZSM-5(35), (**b**) Silicalite-1 (**c**) purity ratio of NO_2_ on HZSM-5(35) and Silicalite-1.

TPD is not only used to evaluate the binding energy interactions of the adsorbate-adsorbent but is also an important index for the assessment of the economic benefits [42]. Figure 9a,b shows the NO_x_ desorption curves of HZSM-5(35) and Silicalite-1 under different RH. TPD curves of NO_x_ on HZSM-5(35) show a single NO (75~85 °C) and two NO_2_ (255~265 °C and 375~395 °C) desorption temperature peaks. More importantly, it is enabled to achieve the recovery of NO_2_ (~62) under an acceptable desorption temperature window for practical applications even at 90% RH, as shown in Figure 9c. Unlike HZSM-5(35), TPD curves of NO_x_ on Silicalite-1 exhibit multimodal distributions with the increase of RH, and the desorption temperature shows an upward trend, with the desorption temperature peaks of NO and NO_2_ mainly distributed at 160~550 °C and 200~525 °C, respectively. The reason is that the smaller pore aperture size is formed in the pore channels or surrounding cation walls of the zeolite framework, leading to a higher desorption activation energy. In particular, it is emphasized that the conversion of NO is completely inhibited by H_2_O(g) at 90%RH, and such a poor purity ratio of NO_2_ for Silicalite-1 would be eliminated. 

### 3.5. Regeneration Performance Tests

**Figure 10:**
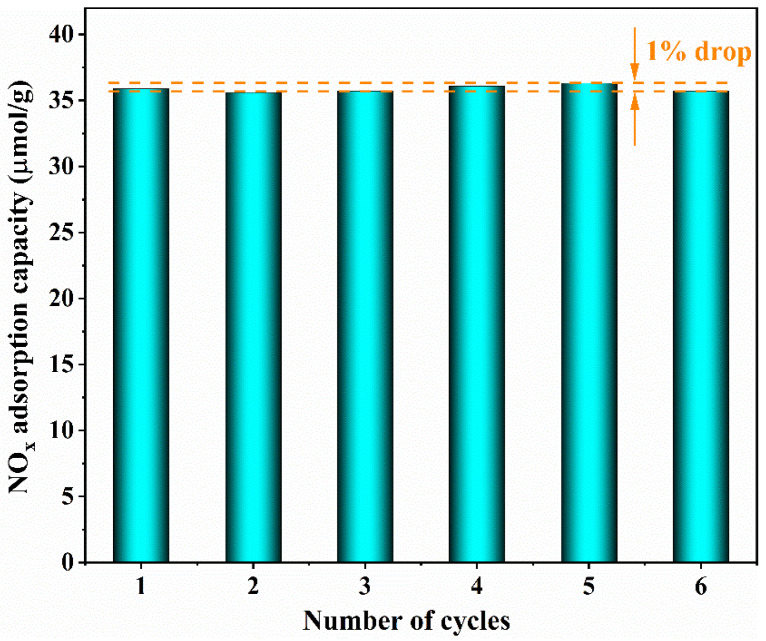
Regeneration cycles of NO_x_ on HZSM-5(35) at 90% RH.

The regeneration tests of NO_x_ are conducted under 90% RH. The NO_x_ adsorption capacity is 99% of the first cycle after six consecutive cycles of use, with a 1% decrease in NO_x_ adsorption capacity, as depicted in Figure 10. Moreover, it can also be observed that the color of HZSM-5(35) changes from white to reddish brown for the adsorption process and returns to white again for the desorption process by naked-eye observation. The robust regeneration performance can provide the possibility of large-scale removal of NO_x_ and other pollution gases. 

## 4. Conclusions

A detailed compilation of the effect of Si/Al ratio on NO_x_ adsorption/desorption behavior was studied on MFI zeolites under different RH. As shown, HZSM-5(35) exhibited the highest adsorption capacity of NO_x_, up to 297.8 μmol/g and 35.4 μmol/g under dry and 90% RH conditions, which was greater than that of Silicalite-1 with barely 59.2 μmol/g and 8.7 μmol/g, respectively. Hence, a novel water-resistance strategy has been proposed for NO_x_ adsorption containing competitive gases including H_2_O(g) and CO_2_. The presence of O_2_ was an essential factor in enhancing NO_x_ adsorption with the optimal 14% O_2_ concentration screened. The effect of CO_2_ on NO_x_ adsorption was relatively small with only a 5.2% reduction even at concentrations up to 10.5%. The results indicated that higher temperature (55 °C) inhibited NO_x_ adsorption (~12.6 μmol/g) with a 64.4% decrease compared to lower temperature (25 °C). TPD curves of HZSM-5(35) exhibited an acceptable industrial desorption temperature window with the main NO_2_ desorption temperature mainly located in the range of 255~265 °C. In contrast, the multimodal NO_x_ desorption temperature peaks of Silicalite-1 are shown with the NO and NO_2_ mainly distributed at 160~550 °C and 200~525 °C, respectively. Six regeneration tests were conducted with only a 1% decrease. This work provides a rational selection strategy of NO_x_ adsorbents in industrial applications.

## Figures and Tables

**Table 1:** Textural properties of MFI zeolites with different Si/Al ratios.

Samples	S_BET_ (m^2^/g)	S_micro_ (m^2^/g)	S_external_ (m^2^/g)	S_meso_ (m^2^/g)	V_total_ (cm^3^/g)	V_micro_ (cm^3^/g)	V_meso_ (cm^3^/g)	D_p_ (nm)
ZSM5(35)	336.9	266.9	70.0	31.9	0.19	0.14	0.05	0.55
ZSM5(110)	372.4	285.2	87.2	43.7	0.22	0.14	0.08	0.55
ZSM5(360)	385.1	290.5	94.6	45.4	0.24	0.15	0.09	0.52
Silicalite-1	413.8	282.6	131.2	58.1	0.28	0.17	0.11	0.52

**Table 2:** Summary of the dynamic adsorption properties, roll-up factor, and efficiency loss factor of NO_x_ on MFI zeolites at 25 °C under different RH conditions.

Samples	RH (%)	Breakthrough Time (s)	Breakthrough Adsorption Capacity (μmol/g)	Saturation Adsorption Capacity (μmol/g)	Roll-Up Factor-*η*	Efficiency Loss Factor-*γ* (%)
HZSM-5 (35)	0	17,775	285.7	297.8	1.0	0
	20	6762	105.1	121.3	5.9	59.3
	40	3663	56.3	68.8	6.3	76.9
	60	3068	48.1	58.2	7.3	80.5
	80	2380	36.3	38.2	8.3	87.2
	90	2167	34.8	35.4	9.6	88.1
HZSM-5 (110)	0	12,020	190.0	206.8	1.0	0
	20	5535	88.9	91.1	3.4	56.0
	40	2942	44.2	56.6	4.6	72.6
	60	2190	34.3	40.8	5.9	80.2
	80	1861	28.8	31.7	6.2	84.7
	90	1695	26.2	28.5	6.4	86.2
HZSM-5 (360)	0	6190	89.3	96.5	1.0	0
	20	2227	35.2	38.4	2.8	60.2
	40	1543	23.3	27.5	3.0	71.5
	60	1200	18.2	21.6	4.2	77.6
	80	925	14.0	16.4	4.5	83.0
	90	750	11.6	13.8	4.8	85.7
Silicalite-1	0	504	7.6	59.2	1.0	0
	20	432	6.1	27.6	1.0	53.4
	40	0	0	18.5	1.0	68.8
	60	0	0	13.2	1.0	77.8
	80	0	0	10.7	1.0	81.9
	90	0	0	8.7	1.0	85.3

## Data Availability

Data will be made available upon reasonable request.
